# Poverty, dirt, infections and non-atopic wheezing in children from a Brazilian urban center

**DOI:** 10.1186/1465-9921-11-167

**Published:** 2010-12-01

**Authors:** Mauricio L Barreto, Sergio S Cunha, Rosemeire Fiaccone, Renata Esquivel, Leila D Amorim, Sheila Alvim, Matildes Prado, Alvaro A Cruz, Philip J Cooper, Darci N Santos, Agostino Strina, Neuza Alcantara-Neves, Laura C Rodrigues

**Affiliations:** 1 Instituto de Saúde Coletiva, Universidade Federal da Bahia, Salvador, Brazil; 2 Departamento Medicina Social, Universidade Federal de Pernambuco, Recife, Brazil; 3 Departamento de Estatística, Universidade Federal da Bahia, Salvador, Brazil; 4 ProAR, Faculdade de Medicina, Universidade Federal da Bahia, Salvador, Brazil; 5 Colegio de Ciências de la Salud, Universidad San Francisco de Quito, Quito, Ecuador; 6 Instituto de Ciências da Saúde, Universidade Federal da Bahia, Salvador, Brazil; 7 London School of Hygiene and Tropical Medicine, London, UK

## Abstract

**Background:**

The causation of asthma is poorly understood. Risk factors for atopic and non-atopic asthma may be different. This study aimed to analyze the associations between markers of poverty, dirt and infections and wheezing in atopic and non-atopic children.

**Methods:**

1445 children were recruited from a population-based cohort in Salvador, Brazil. Wheezing was assessed using the ISAAC questionnaire and atopy defined as allergen-specific IgE ≥0.70 kU/L. Relevant social factors, environmental exposures and serological markers for childhood infections were investigated as risk factors using multivariate multinomial logistic regression.

**Results:**

Common risk factors for wheezing in atopic and non-atopic children, respectively, were parental asthma and respiratory infection in early childhood. No other factor was associated with wheezing in atopic children. Factors associated with wheezing in non-atopics were low maternal educational level (OR 1.49, 95% CI 0.98-2.38), low frequency of room cleaning (OR 2.49, 95% CI 1.27-4.90), presence of rodents in the house (OR 1.48, 95% CI 1.06-2.09), and day care attendance (OR 1.52, 95% CI 1.01-2.29).

**Conclusions:**

Non-atopic wheezing was associated with risk factors indicative of poverty, dirt and infections. Further research is required to more precisely define the mediating exposures and the mechanisms by which they may cause non-atopic wheeze.

## Background

An estimated 300 million people have asthma worldwide; the prevalence has increased over the past 40 years or so among children in industrialized countries [1] and has increased more recently in some developing countries [2]. Asthma is frequently described as an allergic disease, but this does not reflect the complexity of current evidence: although allergic children are more likely to develop asthma, a large proportion of children with asthma do not appear to be allergic. The International Study of Asthma and Allergic diseases in Childhood (ISAAC), an international survey of the prevalence of asthma and other allergic diseases that used a standardized questionnaire and measures of atopy found that the population fraction of asthma attributable to atopy differed greatly between countries by economic development: 40.7% in study centers from 'affluent' countries and 20.3% in centers from 'non-affluent' countries [3]. The prevalence of reported asthma symptoms is as high in urban centers in Latin America as in the UK and the USA [4], but the proportion attributable to atopy is low [5,6].

**Figure 1:**
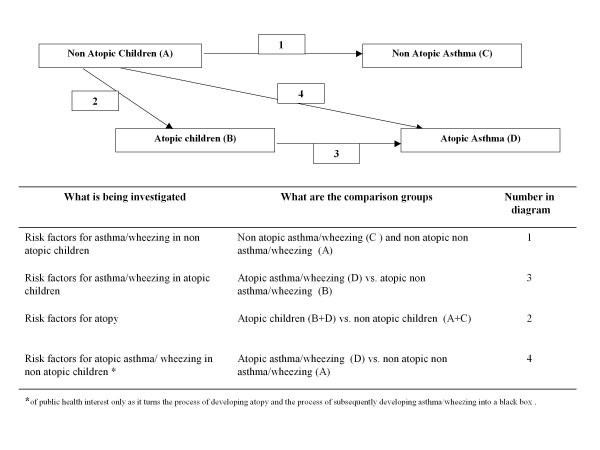
**Conceptual framework for development of asthma/wheezing, with and without atopy, and choice of appropriate comparison groups**.

The causation of asthma is poorly understood. Risk factors reported for asthma as a whole, and for atopic and non-atopic asthma vary between studies and it is likely therefore that the causal mechanisms that underlie these two asthma phenotypes may differ also. Given the increase in the prevalence of asthma among atopic children, understanding why some children become atopic may also contribute to identification of new prevention measures. Very few studies have investigated separately risk factors associated with atopic and non-atopic asthma in the same population [6-13]. A problem in interpreting these studies is that they are not consistent in the choice of comparison groups: approaches used include, among others, comparing both types of asthma with non asthmatic children (irrespective of their atopic status), comparing both groups to non atopic non asthmatic children, or comparing atopic asthmatics with atopic children without asthma, and non atopic asthmatics with non atopic children without asthma. A table summarizing the findings of these studies and the methodological approach they adopted is presented in Table 1. Different choices of comparison groups of course implicitly reflect different study questions and lead to different results. To contribute to the improvement in the design and analysis of population-based studies in this field and to allow studies to be compared more easily, we propose a solution to this conundrum: to be explicit about which question is answered by choosing different comparison groups, and to use the comparison group that is more informative. We propose a framework which makes explicit the assumptions used in the analysis and therefore a better interpretation of findings, enabling the understanding of the complex relationships between atopy, atopic asthma and non atopic asthma (Figure 1). According to this framework, the most informative approach is to investigate separately risk factors for asthma among non-atopic children and those for asthma among atopic children, and risk factors for being atopic in the overall population. We have applied this strategy in the present study to investigate the relationships between risk factors associated with poverty, dirt and infections and wheezing in atopic and non-atopic children and separately for atopy in urban Brazil.

**Table 1:** Summary of the findings of previous studies that analyse in the same population risk factors for atopic or non atopic asthma/wheezing in children

Author, outcome, age group and study sample	Studied Variables	Atopic asthma/wheezing			Non atopic asthma/wheezing	
		**Atopic asthma vs atopic non-asthma**	**Atopic asthma vs non-asthma irrespective of atopy**	**Atopic asthma vs non-atopic non-asthma**	**Non-atopic asthma vs non-atopic non-asthma**	**Non-atopic asthma" vs non-asthma irrespective of atopy**
		**OR (95% CI)**	**OR (95% CI) or mean (sd) when indicated**	**OR (95% CI)**	**OR (95% CI)**	**OR (95% CI) or mean (sd) when indicated**

Rönmark et al, 1999 (7)	Male gender					1.62 (1.03-2.54)
"ever asthma" in 7-8 year olds 3,141 participants (2,149 skin tested)	Family history of asthma		2.95 (1.81-4.81)			3.63 (2.33-5.66)
	Dampness at home					1.78 (1.10-2.89)
	Mother smoker					1.67 (1.04-2.68)
	Breast-feeding <3 months					1.80 (1.11-2.92)
	Pets at home		0.60 (0.36-0.98)			

Braun-Fahrländer et al, 2002 (20) asthma in 6-13 year olds	Endotoxin load	0.52 (0.25-1.07)^c^			1.00 (0.46-2.21)^e^	
	(units/m^2 ^of mattress surface)	0.64 (0.33-1.25)^d^			1.82 (1.04-3.18)^f^	
812 participants	Prevalence of outcome according to farming status					
	Farming	3.1 (1.2-5.0)^g^			1.6 (0.2-2.9)^i^	
		4.7 (2.4-7.0)^h^			1.6 (0.2-2.9)^l^	
	Non farming	5.9 (3.8-8.0)^g^			2.6 (1.2-5.0)^i^	
		5.9 (3.8-8.0)^h^			6.1 (4.0-8.2)^l^	

Priftanji et al, 2002 (8)	Mother had asthma		2.45 (1.06-5.66)			
asthmatic symptoms in 20-44 year olds	Mother smoked					2.66 (1.35-5.26)
717 participants	Subject ever smoked					1.98 (1.17-3.35)

García-Marcos et al, 2005 (9) current wheezing in 9-12 year olds 2,720 participants	Male gender	1.53 (1.09-2.14)				
	Paternal or maternal asthma	2.16 (1.44-3.22)			1.74 (1.17-2.58)	
	Mother smoker (1^st ^year)				2.66 (1.13-6.25)	
	Mould stains currently					

Kelley et al, 2005 (10) asthma in 6-16 year olds	Mean PIR (Poverty/Income ratio)^m^		2.0 vs 2.2 (P < 0.5)			
5,244 participants	Mean education level of adult respondent (years)		12.4 vs 12.0 (P < 0.5)			12.2 vs 12.0 (P < 0.5)
	Mean BMI percentile					68.5 vs 57.7 (P < 0.5)

Janson et al, 2007 (11) asthma, 13-14 year olds	BMI (per 5 units increase)		1.62 (1.06-2.48)			1.55 (1.02-2.36)
901 participants	Parental asthma		2.16 (1.12-4.18)			2.35 (1.23-4.52)
	Early life infections					
	Otitis (≥5/year)					1.99 (1.02-3.88)
	Croup					2.80 (1.44-5.43)
	Cat or dog as infant					2.17 (1.16-4.04)
	Window pane condensation					2.45 (1.11-5.40)
	Dirty school					2.50 (1.28-4.89)

De Meer et al, 2009 (12) recent wheeze in 8-13 year olds 1,547 participants	High parental education			0.89 (0.60-1.31)	0.65 (0.43-0.99)	

Kurukulaaratchy et al, 2010 (13) wheeze at 10 years of age 1,373 participants (1,036 skin tested)	Maternal asthma				4.08 (1.85-9.00)	
	Recurrent chest infection at 2 years				3.99 (1.78-8.92)	
	Sibling asthma			2.10 (1.04-4.23)		
	Eczema at 1 year			2.80 (1.01-7.80)		
	Rhinitis at 4 years			4.74 (1.61-13.47)		
	Male gender			2.73 (1.36-5.48)		

Moncayo et al, (6) recent wheeze in 6-16 year olds 3,960 participants (3,821 skin tested)	Male gender			2.73 (1.44-5.16)		
	Heavy *T. trichiura *infection			0.24 (0.09-0.63)		
	Watching TV > 3 hrs/day				1.51 (1.06-2.16)	
	Maternal allergic diseases				3.24 (2.42-4.32)	
	Birth order ≥4				0.71 (0.57-0.88)	
	Age ≥ 13 years				0.39 (0.25-0.62)	

^a ^OR (95% CI) for the occurrence of atopic asthma

^b ^OR (95% CI) for the occurrence of non-atopic asthma

^c ^OR (95% CI) for the occurrence of atopic asthma with increase of endotoxine load from the lowest quartile to the highest quartile (children non farming households)

^d ^OR (95% CI) for the occurrence of atopic wheeze with increase of endotoxine load from the lowest quartile to the highest quartile (children non farming households)

^e ^OR (95% CI) for the occurrence of non-atopic asthma with increase of endotoxine load from the lowest quartile to the highest quartile (children non farming households)

^f ^OR (95% CI) for the occurrence of non-atopic wheeze with increase of endotoxine load from the lowest quartile to the highest quartile (children non farming households)

^g ^atopic asthma

^h ^atopic wheeze

^i ^non-atopic asthma

^l ^non-atopic wheeze

^m ^ratio of income to the family's appropriate poverty threshold (US Census Bureau).

## Methods

### Study area, population and design

This study is part of a cohort study of asthma and allergies conducted in Salvador, a large city in Northeast Brazil with a high prevalence of wheezing reported in the ISAAC surveys [14]. Methods have been described in details elsewhere [15]. Briefly, the study population consists of a cohort of 1,445 children previously studied to evaluate the impact of a sanitation program and enrolled when they were 0-3 years old in the period 1996 to 2003 [16-18]. This first study was originally designed to enroll three separate cohorts of children aged 0-3 years recruited in 1997, 2001 and 2003, respectively, from 24 small sentinel-areas selected to represent the population without sanitation in Salvador [19]. For each cohort, children were randomly selected among those living in the areas. Sampling and data collection procedures and instruments were the same in the 3 surveys and follow-up, thus making the study populations comparable. Within each selected area, a survey was conducted at baseline and each cohort was then followed-up.regularly for a period of one year from recruitment. During follow-up, children were visited every 2 weeks and data on occurrence of diarrhoea, cough, shortness of breath and fever as reported by the guardian were collected. To take advantage of these three cohorts (since detailed information for them had been collected on early life factors proposed as potential risk factors for asthma and other allergic diseases), the children who were aged 4-11 years and had complete follow-up information were selected for the present study.

The present analysis used outcomes (wheezing and atopy) and exposures measured in 2005 when children were aged 4 to 11 years (demographic and socioeconomic factors, environmental factors, maternal and family factors) and other exposures measured earlier during the first year of follow-up (days of illness with respiratory infections and diarrhea in the first years of life).

### Data Collection

Information was collected, either in the earlier cohort or in 2005, on i) demographic and socioeconomic factors: age, sex, monthly income, maternal schooling, number of electric appliances; ii) environmental factors: sanitation and water supply, electricity, presence of household pets, presence of pets and animals outside household, contact with animals in farms, housing (type of construction), cooking materials; iii) maternal and family factors: maternal smoking during pregnancy and in the first year of life, presence of other household smokers, maternal allergic diseases, duration of breast-feeding, day care attendance, presence of other siblings, birth order, BCG scar and anti-helmintic treatment; iv) exercise/sedentarism: daily hours watching television and frequency of vigorous exercise; v) nutritional factors: Body Mass Index; v) infections: reported incidence of respiratory infection and diarrhea during the first year of follow up. We decided to restrict our analysis to those with at least 90 days of follow-up and classified as low frequency a proportion of days with respiratory illness (ARI) and diarrhea between 1 to 7 days of the follow-up. Day with diarrhea was defined as the report of 3 or more liquid or semi-liquid motions, and day with respiratory illness (ARI) was defined by the simultaneous report of cough, fever and shortness-of-breath in a 24 hours period.

The ISAAC questionnaire, translated into Brazilian Portuguese [20], was applied to parents during household visits. Presence of wheeze was defined by the question, "Have you had wheezing or whistling in the chest in the past 12 months?" All 1,445 children had blood collected for serology to *Toxoplasma gondii*, *Helicobacter pylori*, Herpes simplex virus (HSV), Varicella zoster virus (VZV), Epstein-Barr virus (EBV), Hepatitis A (HAV) and *Ascaris lumbricoides *and for levels of allergen specific IgE to *Dermatophagoides pteronyssinus*, *Blomia tropicalis*, *Blattella germanica *and *Periplaneta americana *(ImmunoCAP assay, Pharmacia, Uppsala, Sweden). Children were classified as atopic if the level of IgE to any of the 4 allergens was >0.70 kU/L. These four specific mite and cockroach allergens were chosen to measure atopy based on the findings of positivity to allergen skin prick tests to a panel of seven relevant aeroallergens. Positive skin prick tests to the four allergens accounted for 99% of all children with positive tests (389 out of 394). Based on atopy status and report of wheezing children were classified into 4 categories: atopic wheezers, non-atopic wheezers, atopic non-wheezers, and non-atopic non-wheezers.

### Laboratory examinations

HSV, VZV, EBV, *T. gondii *and *H. pylori *infections were diagnosed by detecting specific serum IgG using commercially available kits (Diamedix, Miami, FL, USA). Presence of anti-HAV IgG was determined using kits from ADALTIS (Toronto, Canada). This assays cut-offs were determined following the suppliers' directions, i.e., an index value obtained by the ratio Absorbance of sample/Absorbance of a calibrator (a solution containing human serum with moderate reactivity). A ratio value greater than 1.00 was considered positive. Anti-*A. lumbricoides *IgG4 was detected by indirect ELISA. Details of the technique were presented elsewhere [21].

### Statistical Analysis

We investigated separately risk factors for asthma among non-atopic children, among atopic children, and for being atopic in the overall population, following the conceptual framework, and using the comparison groups described in Figure 1. We used bivariate and multivariate multinomial logistic regression [22] to identify separately risk factors for wheezing among non-atopic children (comparing non-atopic wheezers with non-atopic non-wheezers) and risk factors for wheezing among atopic children (comparing atopic wheezers with atopic non-wheezers). We used multinomial logistic regression because it treats the categories of the polytomy (atopic wheezers, non-atopic wheezers, atopic non-wheezers, and non-atopic non-wheezers) in a non-arbitrary order and also addresses several sets of log-odds, corresponding to different dichotomies. In addition to this, polychotomous logistic regression allows the construction of statistical significance tests of the variables accounting for simultaneous differences in probabilities of all the response categories, and not just individual groups. A separate analysis was done using ordinary logistic regression to study risk factors for atopy, comparing atopic children with non-atopic children, irrespective of wheeze. A backward elimination procedure using likelihood ratio test (p-value < 10%) was adopted for all analyses to remove variables individually. Analyses for potential risk factors, apart from respiratory symptoms, were done with 1,309 children for whom complete data were available. Analyses were done using STATA, version 10 (StataCorp, College Station, TX, USA).

### Ethics

Ethical approval was obtained from the Brazilian National Ethical Committee and from the LSHTM. Written informed consent was obtained from the legal guardians.

## Results

Among the 1,309 children - 384 (29.3%) reported wheezing in the last 12 months and 486 (37.1%) had specific IgE in the serum above 0.70 kU/L for at least one of the tested allergens. Children were classified into 4 groups: non-atopic non-wheezers (613 children), atopic non-wheezers (312), non-atopic wheezers (210), and atopic wheezers (174). The prevalence of wheezing was 34.8% among atopic children, while this prevalence was 25.0% among non-atopic children. Atopic children were 60% more likely to wheeze than non-atopic children with OR = 1.60 (1,26; 2.04).

Table 2 presents description of the demographic, social and environmental variables for each group classified based on atopy and wheezing. Some of these distributions does not seem to change across the groups, like child's sex and maternal education. However, a higher prevalence of parental asthma is verified among non-atopy wheezers and atopic wheezers (21.4% and 17.4%, respectively). Prolonged respiratory infection (≥8 days) in early childhood is also observed in these groups (prevalences of 11.0% and 10.0%, respectively). At the same time, a greater proportion of atopic (non-wheezers and wheezers) children had detectable values for IgG4 (19.4% and 22.4%) compared to non atopics (non-wheezers and wheezers) children (12.7% and 16.2%, respectively). A table listing the results with all investigated variables is available in the Additional file 1, Table S1.

**Table 2:** Distribution of potential risk factors according to atopy-wheezing groups in 1,309 children.

	Non atopic			Atopic		
	
Potential risk factors	overall	non-wheezers	wheezers	overall	non-wheezers	wheezers
	(N = 823)	(N = 613)	(N = 210)	(N = 486)	(N = 312)	(N = 174)
Social/familial and Demographic factors						

Sex						
Female	46.1	46.2	45.7	49.0	51.3	44.8
Male	53.9	53.8	54.3	51.0	48.7	55.2

Age						
<5	36.1	31.0	51.0	33.7	28.8	42.5
6-7	35.5	37.0	31.0	35.8	37.2	33.3
>8	28.4	32.0	18.0	30.5	34.0	24.1

Mother education						
Elementary education	21.9	20.6	25.7	22.4	21.2	24.7
More than elementary education	47.3	48.1	50.5	35.8	48.4	45.4
More than high school	29.4	31.3	23.8	30.2	30.4	29.9

Parental Asthma						
No	85.4	87.8	78.6	88.9	92.3	82.8
Yes	14.6	12.2	21.4	11.1	7.7	17.2

Dirt and Infections						

Routine cleaning in all the rooms						
No	4.7	3.4	8.6	3.3	2.2	5.2
Yes	95.3	96.6	91.4	96.7	97.8	94.8

Having attended day care						
No	83.6	85.8	77.1	84.6	84.3	85.1
Yes	16.4	14.2	22.9	15.4	15.7	14.9

Rodents in the household						
No	41.4	44.5	32.4	44.7	43.3	47.1
Yes	58.6	55.5	67.6	55.3	56.7	52.9

*Tgondi*						
No	80.1	80.6	78.6	84.8	86.2	82.2
Yes	19.9	19.4	21.4	15.2	13.8	17.8

*IgG4*						
No detectable	86.4	87.3	83.8	79.4	80.4	77.6
Detectable	13.6	12.7	16.2	20.6	19.6	22.4

Respiratory symptoms						
No	68.3	72.1	56.7	69.2	73.2	60.8
For 1-7 days	26.6	24.7	32.3	24.3	21.9	29.2
For ≥8 days	5.1	3.2	11.0	6.5	4.9	10.0

Diarrhoea						
No	24.8	26.1	20.7	23.0	23.5	22.1
For 1-7 days	43.9	45.6	38.7	47.5	49.0	44.3
For ≥8 days	31.3	28.3	40.6	29.5	27.5	33.6

Table 3 presents adjusted associations between the demographic, social and environmental variables. This table presents results related to the adjustment of two models: the multinomial logistic regression (results in the second and third columns) and binary logistic regression for atopy (results in the fourth column). For the multinomial logistic regression, we only presented those results that discriminate risk factors for atopic and non-atopic/wheezing phenotypes. Variables are shown that were meaningfully associated with at least one outcome either in univariate or multivariate analysis. Crude associations obtained from univariate regression models are presented in Additional file 1, Table S1. The directions of the associations did not change considering crude and adjusted measures, and the changes of magnitude of these associations were generally modest. The lack of significance observed for some associations likely reflects lack of power. In general, parental asthma and frequent respiratory infections in childhood increased the risk of wheeze in both atopic and non-atopic children. Factors associated with wheeze only in non-atopic children were mainly related to dirt, infections or poverty: infrequent household cleaning, household rodent infestations, having attended day care and having had frequent respiratory infections and diarrhea in childhood and low maternal educational level. No factors were associated with wheeze only in atopic children. The only factor that was associated with a meaningful increase in atopy was the presence of IgG4 to *A lumbricoides*, a marker of past or active ascariasis.

**Table 3:** Multivariate analysisa of potential risk factors related to development of atopic and non-atopic wheezing and atopy in 1,309 children

Potential risk factors	**Non-atopic wheezers (vs. non-atopic non-wheezers)**^**b**^	**Atopic wheezers (vs. atopic non-wheezers)**^**b**^	**Atopic (vs. non-atopics)**^**c**^
	
	OR (95%CI)	OR (95%CI)	OR (95%CI)
Social/familial factors			

Mother education (reference group: High)			
Medium	1.30 (0.88 to 1.93)	0.94 (0.60 to 1.46)	0.95 (0.73 to 1.24)
Low(elementary or illiterate)	1.49 (0.93 to 2.38)	1.24 (0.73 to 2.11)	1.01 (0.73 to 1.39)

Parental Asthma (reference: no parental asthma)	1.91 (1.25 to 2.92)	2.64 (1.48 to 4.73)	0.73 (0.51 to 1.03)

Dirt and Infections			

Infrequent household cleaning (reference: frequent cleaning)	2.49 (1.27 to 4.90)	2.27 (0.82 to 6.29)	0.72 (0.39 to 1.31)

Rodents in home (reference: no rodents)	1.68 (1.21 to 2.34)	0.79 (0.54 to 1.15)	0.90 (0.71 to 1.13)

Having attended day care (reference: no attendance)	1.52 (1.01 to 2.29)	0.83 (0.49 to 1.41)	0.92 (0.67 to 1.26)

*T. gondii *seropositivity (reference: negative)	1.23 (0.82 to 1.84)	1.51 (0.90 to 2.54)	0.68 (0.50 to 0.93)

*A. lumbricoides*-specific IgG4 (reference: negative)	1.25 (0.79 to 1.97)	1.20 (0.75 to 1.92)	1.73 (1.27 to 2.34)

Respiratory symptoms (reference: no symptoms)			
for 1-7 days	1.54 (1.01 to 2.36)	1.54 (0.92 to 2.59)	0.89 (0.65 to 1.21)
for ≥8 days	4.87 (2.26 to 9.76)	2.27 (0.97 to 5.38)	1.31 (0.74 to 2.31)

Diarrhea (reference: no diarrhoea)			
for 1-7 days	1.09 (0.67 to 1.80)	1.02 (0.58 to 1.81)	1.18 (0.85 to 1.64)
for ≥8 days	1.59 (0.95 to 2.66)	1.29 (0.70 to 2.40)	1.03 (0.72 to 1.49)

^a ^controlled by age and sex, and adjusted for all factors included in the table.

^b ^multinomial logistic regression

^c ^ordinary logistic regression

## Discussion

The present study showed many differences in risk factors for wheezing in atopic and non-atopic children. Common risk factors for the two wheeze phenotypes were parental asthma (with an effect of similar magnitude in atopic and non-atopic wheezers), and to a lesser degree respiratory infections in early childhood (with a weaker effect in atopic children). No other studied factor was meaningfully associated with wheezing in atopic children, but several factors were associated with wheezing in non-atopic children. These were related to poverty and dirt (lower maternal educational level, lower frequency of routine room cleaning, reported presence of rodents in or around the house) and having attended day care, a potential marker of greater contact between young children and greater infectious exposures during childhood. The only factors associated with atopy - measured as level of allergen specific IgE above 0.70 kU/L - were seropositivity (IgG) to *T. gondii *(lowering the risk) and the presence of IgG4 to *A. lumbricoides *(increasing the risk).

There is an extensive literature on risk factors for asthma/wheezing, but very often studies do not discriminate between atopic and non-atopic asthma/wheezing phenotypes. The scarce literature that discriminates between risk factors for the two phenotypes is difficult to interpret because of differences in comparison groups used. This is a problem because the choice of comparison groups defines the study question. Briefly, as presented in Table 1, studies of risk factors for atopic asthma/wheezing have used three different comparison groups: (i) atopic with no asthma/wheezing [7,9,23], (ii) non-atopic with no asthma/wheezing [6,12,13], and (iii) no-asthma/wheezing irrespective of atopy [8,10,11]. The first comparison group, which we used in our study, correctly identified risk factors for the development of asthma/wheezing in atopic children. The risk estimated when the second group is used includes both risks for development of atopy and for development of asthma/wheezing in atopic children, although not helpful in understanding causation could have a public health purpose. The use of the third group generates results not really interpretable as the presence of atopic and non-atopic children in the comparison group generates an arbitrary combination of risks for development of asthma/wheezing in atopic children and risks for the development of atopy. Two different comparison groups have been used to study risk factors for non-atopic asthma/wheezing: non-atopic with no asthma/wheezing [7,10,13,23] and children with no asthma irrespective of atopy [8,9,11,12 ]. The first comparison group, which we used in our study, correctly identified risk factors for the development of asthma/wheezing in non-atopic children. The second group generate results that are difficult to interpret because they merge risks for the development of asthma/wheezing in non-atopic children and protection against the development of atopy.

Taking these issues into account, epidemiological studies reported to date have identified respiratory infections in early childhood [10,13,24], family history of asthma [6-9,11,13] and smoking in the household [7-9,12] to be more consistently associated with non-atopic asthma. Several of these studies have reported factors that while not reproduced consistently, can be interpreted as being related to different aspects of poverty, infection and dirt (parental education, dampness, mould stains, dirt school, pets, and endotoxin exposure) [7,8,11,12,23].

While there is evidence that atopy may increase with socioeconomic status [25,26], the relationship between socioeconomic status and asthma/wheezing is more complex [27]. Within Europe, asthma/wheezing used to be more common among individuals of high socioeconomic status and that this trend may have reversed in recent years [28,29]. In the USA, asthma/wheezing is clearly more frequent in poor inner city populations although there is no evidence to clarify whether this is attributable to a greater prevalence or poor asthma control because of more limited access to health care [30,31]. Studies in urban environments in Latin America have reported a very high prevalence of wheezing among children living in poor neighborhoods [32]. A recent ecological analysis of survey data using population samples from 64 countries has described a bimodal asthma distribution with a higher prevalence in low-income and high-income countries, and lower prevalence in middle-income countries [33]. Conflicting results may have resulted from a failure to discriminate atopic from non-atopic asthma/wheezing. There are indications that markers of dirt may have opposite effects in the atopic and non-atopic phenotypes [34]. It is essential that further studies investigate separately atopic and non-atopic asthma/wheezing.

There are plausible mechanisms by which poverty may increase the prevalence of non-atopic asthma/wheezing. In one study, reduced exposure to second-hand cigarette smoke explained a significant proportion of the protection against non-atopic wheezing in highly educated families [12]. Respiratory infections [35] and dirt [36] are also more frequent among the poorest. There is an interesting ongoing discussion about a role for exposure to endotoxin, a highly immunologically active constituent of cell walls of gram-negative bacteria - as the mechanism by which 'dirt' may induce the inflammatory effects in the lungs that could underlie non-atopic asthma. This has been proposed as a potential mechanism for the lower prevalence of asthma/wheezing and allergic rhinitis in rural areas of Europe [30]. There is evidence that high-level exposures to endotoxin can have a protective effect on atopic asthma/wheezing but may increase the risk of non-atopic asthma/wheezing [23,34,37]. Thus, microbial exposures may somewhat paradoxically be a protective factor for atopic asthma/wheezing but an important risk factor for non-atopic asthma/wheezing. Attending day care is associated with an increased risk of respiratory infections [38].

The present study may have lacked power to detect some associations because of loss of power by separation of children in atopic and non-atopic wheezers. However, this separation is one of the strengths of the study. Other strengths are: (i) allergen specific IgE was measured for all children and not restricted to wheezers; (ii) risk factors for atopic and non-atopic wheeze were studied in the same population, and multinomial logistic regression analysis was used to maximize the value of using the same population; (iii) analysis was informed by a conceptual framework, and therefore used the appropriate comparison group to study risk factors for wheezing in atopic and non-atopic children; (iv) early and late potential risk factors were measured concurrently, avoiding recall bias; and (v) is the first study to assess separately risk factors for atopic and non-atopic wheezing in an urban context in a developing country.

In conclusion, parental asthma and early life respiratory infections increased the risk of wheezing in atopic and non-atopic children, but other factors were associated only with non-atopic wheezing. These factors were associated with poverty, dirt and infections: low maternal educational level, infrequent cleaning of the house, household rodent infestation, and having attended day care. There is a need for further research into risk factors for asthma that investigate separately atopic and non-atopic individuals using appropriate comparison groups as indicated in the proposed framework. Such studies should explore further the relationship between socioeconomic status and non-atopic wheezing to clarify which aspects of poverty and dirt mediate the associations observed.

## Conflict of interests

The authors declare that they have no competing interests.

## Authors' contributions

MLB and LCR participated in the study design in the interpretation of data, and drafted the manuscript; SSC, AAC, PJC, DNS, NA-N, RF, RE and LDA contributed to the analysis, interpretation and drafting of the manuscript; SA, MP, AS contributed to the data collection, analysis, interpretation and drafting of the manuscript. All authors read and approved the final manuscript.

## Supplementary Material

Additional file 1 **Table S1**. Univariate association of socioeconomic, demographic, environmental and personal factors with wheezing with and without atopy in 1,309 children. Click here for file
