# Supplementary material for: Poverty, dirt, infections and non-atopic wheezing in children from a Brazilian urban center
Source: Respir Res. 2010 Dec 1;11(1):167. doi: 10.1186/1465-9921-11-167 (PMC3002921; doi:10.1186/1465-9921-11-167)
Supplement: Additional file 1 — Table S1. Univariate association of socioeconomic, demographic, environmental and personal factors with wheezing with and without atopy in 1,309 children. [file 1465-9921-11-167-S1.DOC]

Poverty, dirt and infections as risk factors for non-atopic wheezing in children from a Brazilian urban center

Mauricio L Barreto, Sergio S Cunha, Rosemeire Fiaccone, Renata Esquivel, Leila D Amorim, Sheila Alvim, Matildes Prado, Alvaro A Cruz, Philip J Cooper,Darci N Santos, Agostino Strina, Neusa Alcantara-Neves, Laura C Rodrigues

Table S1: Association of socioeconomic, demographic, environmental and personal factors with wheezing with and without atopy in 1,309 children

|  |  | Non-atopic wheezing  (vs non-atopic without wheezing) |  |  | Atopic wheezing  (vs atopic without wheezing) |
| --- | --- | --- | --- | --- | --- |
|  | N (%) | OR (95%CI) |  | N (%) | OR (95%CI) |
| Social/familial and Demographic factors |  |  |  |  |  |
|  |  |  |  |  |  |
| Gender |  |  |  |  |  |
| Female | 96 (15.6) | 1 |  | 78 (12.6) | 1 |
| Male | 114 (16.6) | 1,02 (0,74;1,39) |  | 96 (13.9) | 1,30 (0,89;1,88) |
| Age group in years |  |  |  |  |  |
| <5 | 107 (23.2) | 1 |  | 74 (16.1) | 1 |
| 6-7 | 65 (13.9) | 0,51 (0,35;0,73) |  | 58 (12.4) | 0,61(0,39;0,94) |
| >8 | 38 (9,9) | 0,34 (0,23;0,52) |  | 42 (11.0) | 0,48(0,30;0,77) |
| Mother’s education |  |  |  |  |  |
| Illiterate or primary | 54 (18,7) | 1 |  | 43 (14.9) | 1 |
| Secondary education | 106 (16.8) | 0,84 (0,57;1,24) |  | 79 (12.5) | 0,80 (0,50;1,29) |
| High school or university | 50 (12.98) | 0,61 (0,39;0,95) |  | 52 (13.4) | 0,84 (0,50;1,40) |
| Parental Asthma |  |  |  |  |  |
| No | 165 (14.5) | 1 |  | 144 (12.7) | 1 |
| Yes | 45 (25.9) | 1,96 (1,30;2,94) |  | 30 (17.2) | 2,50 (1,41;4,43) |
| Number of siblings, except the study child |  |  |  |  |  |
| No other child | 20 (32.8) | 1 |  | 8 (12.70) | 1 |
| One child | 100 (13.8) | 0,35 (0,18;0,65) |  | 91(12.05) | 0,53(0,20;1,42) |
| Two children | 65(16.9) | 0,45 (0,23;0,87) |  | 62(15.58) | 0,80(0,29;2,18) |
| Three or more children | 25(18.0) | 0,45 (0,21;0,95) |  | 18(12.24) | 0,64(0,21;1,96) |
| Mother’s skin color |  |  |  |  |  |
| White | 15 (13.2) | 1 |  | 25 (21.9) | 1 |
| Mixed | 136 (16.3) | 1,13 (0,62;2,09) |  | 105 (12.6) | 0,52 (0,28;0,95) |
| Black | 53 (16.6) | 1,14 (0,59;2,20) |  | 42 (13.1) | 0,58 (0,29;1,13) |
| Environmental factors |  |  |  |  |  |
| Presence of mould in the house |  |  |  |  |  |
| No | 57 (13.8) | 1 |  | 45 (10.9) | 1 |
| Yes | 153 (17.1) | 1,36 (0,96;1,92) |  | 129 (14.4) | 1,45 (0,96;2,20) |
| Infrequent household cleaning |  |  |  |  |  |
| No | 18 (32.7) | 1 |  | 9 (16.4) | 1 |
| Yes | 192 (15.3) | 2,63 (1,39; 5,00) |  | 165 (13.2) | 2,38 (0,87; 6,67) |
| Mother’s smoking (currently) |  |  |  |  |  |
| No | 181 (15.8) | 1 |  | 153 (13.3) | 1 |
| Yes | 29 (18.0) | 1,17 (0,74;1,85) |  | 21 (13.0) | 1,02 (0,58;1,80) |
| Other smoking people in the house |  |  |  |  |  |
| No | 160 (15.7) | 1 |  | 139 (13.6) | 1 |
| Yes | 50 ( 17.4) | 1,08 (0,74;1,56) |  | 35 (12.2) | 0,96 (0,60;1,52) |
| Water source |  |  |  |  |  |
| Piped | 165 (15.1) | 1 |  | 153 (14.0) | 1 |
| Not piped | 45 (20.8) | 1,45 (0,98;2,15) |  | 21 (9.7) | 0,67 (0,39;1,15) |
| Presence of rats in the house |  |  |  |  |  |
| No | 68 (12.2) | 1 |  | 82 (14.7) | 1 |
| Yes | 142 (18.9) | 1,68 (1,21;2,33) |  | 92 (12.3) | 0,86 (0,59;1,24) |
| Presence of cats in the house |  |  |  |  |  |
| No | 164 (15.2) | 1 |  | 133 (12.3) | 1 |
| Yes | 22 (18.5) | 1,35 (0,79;2,29) |  | 21 (17.6) | 1,69 (0,91;3,13) |
| Had in the past | 24 (21.4) | 1,53 (0,91;2,57) |  | 20 (17.9) | 2,12 (1,09;4,11) |
| Presence of dog in the house |  |  |  |  |  |
| No | 121 (15.2) | 1 |  | 97 (12.2) | 1 |
| Yes | 50 (17.5) | 1,11 (0,76;1,63) |  | 40 (14.0) | 1,48 (0,92;2,37) |
| Had in the past | 39 (17.1) | 1,25 (0,82;1,91) |  | 37 (16.2) | 1,39 (0,86;2,26) |
| Garbage collection frequency |  |  |  |  |  |
| Daily or alternate day | 156 (16.3) | 1 |  | 130 (13.6) | 1 |
| Once a week or rarely | 51 (16.0) | 0,98 (0,68;1,41) |  | 40 (12.6) | 0,88 (0,57;1,37) |
| Presence of cockroaches |  |  |  |  |  |
| No | 34 (12.3) | 1 |  | 35 (12.7) | 1 |
| Yes | 176 (17.0) | 1,50(0,99;2,27) |  | 139 (13.5) | 1,13 (0,71;1,78) |
| Day-care attendance |  |  |  |  |  |
| No | 162 (14.7) | 1 |  | 148 (13.5) | 1 |
| Yes | 48 (22.9) | 1,79 (1,21;2,66) |  | 26 (12.4) | 0,94 (0,56;1,58) |
| Excreta disposal (Past/Present) |  |  |  |  |  |
| None | 29 (16.4) | 1 |  | 19 (10.7) | 1 |
| Some (past or present) | 80 (15.0) | 1.05 (0,64;1,71) |  | 75 (14.0) | 1,09 (0,59;2,01) |
| Always | 95 (17.3) | 1,10 (0,68;1,78) |  | 74 (13.5) | 1,34 (0,72;2,49) |
| Number of people sharing child’s bedroom |  |  |  |  |  |
| None | 2 ( 16.7) | 1 |  | 2 (16.7) | 1 |
| Sharing in some point | 12 ( 14.5) | 0,46 (0,07;3,09) |  | 9 (10.8) | 0,98 (0,16;5,99) |
| Always sharing | 196 (16.1) | 0,51 (0,09;3,10) |  | 163 (13.4) | 1,43 (0,28;7,48) |
| Infections |  |  |  |  |  |
| HSV |  |  |  |  |  |
| No | 85 (14.6) | 1 |  | 85 (14.6) | 1 |
| Yes | 124 (17.2) | 1,14 (0,83;1,57) |  | 89 (12.3) | 0,91 (0,63;1,32) |
| EBV |  |  |  |  |  |
| No | 24 (16.3) | 1 |  | 27 (18.4) | 1 |
| Yes | 185 (16.0) | 0,83 (0,50;1,36) |  | 147 (12.7) | 0,74 (0,43;1,26) |
| VZV |  |  |  |  |  |
| No | 120 (17.0) | 1 |  | 90 (12.7) | 1 |
| Yes | 87 (14.8) | 0,82 (0,60;1,13) |  | 83 (14.1) | 1,19 (0,82;1,73) |
| HAV |  |  |  |  |  |
| No | 170 (15.8) | 1 |  | 149(13.8) | 1 |
| Yes | 39 (17.4) | 1,09 (0,73;1,63) |  | 25 (11.2) | 0,80 (0,48;1,33) |
| *Toxoplasma gondii* |  |  |  |  |  |
| No | 165 (15.4) | 1 |  | 143 (13.4) | 1 |
| Yes | 45 (18.9) | 1,13 (0,77;1,66) |  | 31 (13.0) | 1,36 (0,82;2,25) |
| *Helicobacter pilori* |  |  |  |  |  |
| No | 150 (17.0) | 1 |  | 120 (13.6) | 1 |
| Yes | 44 (12.8) | 0,69 (0,47;1,00) |  | 42 (12.2) | 0,87 (0,56;1,33) |
| IgE anti-Ascaris |  |  |  |  |  |
| Negative | 131 (20.1) | 1 |  | 37(5.7) | 1 |
| Positive | 78 (11.9) | 1,16 (0,84;1,61) |  | 137(20.9) | 1,26 (0,81;1,97) |
| IgG4 anti-Ascaris |  |  |  |  |  |
| Negative | 176 (16.0) | 1 |  | 135 (12.3) | 1 |
| Positive | 34 (16.0) | 1,33 (0,86;2,05) |  | 39 (18.4) | 1,19 (0,76;1,87) |
| *Toxocara canis* |  |  |  |  |  |
| No | 107 (16.1) | 1 |  | 84 (12.6) | 1 |
| Yes | 99 (16.2) | 1,12 (0,81;1,53) |  | 86 (14.1) | 0,97 (0,67;1,41) |
| *A. lumbricoides* infection in 2005 and some years early |  |  |  |  |  |
| Negative in both periods | 101 (14.1) | 1 |  | 92 (12.8) | 1 |
| Positive in at least one period | 36 (14.5) | 0,94 (0,61;1,45) |  | 29 (11.7) | 1,07 (0,64;1,78) |
| Positive in both periods | 17 (22.7) | 1,78 (0,95;3,34) |  | 11 (14.7) | 1,48 (0,65;3,36) |
| *Trichuris trichiura* infection in 2005 and some years early |  |  |  |  |  |
| Negative in both periods | 110 (13.8) | 1 |  | 107(13.4) | 1 |
| Positive in at least one period | 29 (16.8) | 1,19 (0,74;1,91) |  | 19 (11.0) | 0,86 (0,48;1,56) |
| Positive in both periods | 15 (22.1) | 1,62 (0,85;3,10) |  | 8 (11.8) | 1,14 (0,46;2,85) |
| Respiratory symptoms, number of days/year |  |  |  |  |  |
| None | 88 (12.9) | 1 |  | 73 (10.7) | 1 |
| 1-7 days | 50 (19.5) | 1,66 (1,11;2,49) |  | 35 (13.7) | 1,61 (0,97;2,66) |
| More than 8 days | 17 (30.4) | 4,40(2,12;9,17) |  | 12 (21.4) | 2,48 (1,07;5,77) |
| Diarrhoea, number of days/year |  |  |  |  |  |
| None | 32 (13.3) | 1 |  | 27 (11.2) | 1 |
| 1-7 days | 60 (13.3) | 1,08 (0,66;1,74) |  | 54 (12.0) | 0,96 (0,55;1,68) |
| More than 8 days | 63 (20.6) | 1,82 (1,12;2,98) |  | 41 (13.4) | 1,30 (0,71;2,36) |
| Vaccines |  |  |  |  |  |
| HIB vaccine |  |  |  |  |  |
| No | 52 (13.9) | 1 |  | 45 (12.0) | 1 |
| 1 or 2 doses | 39 (15.7) | 1,08 (0,68;1,74) |  | 27 (10.8) | 1,00 (0,56;1,80) |
| 3 or more doses | 67 (21.5) | 1,89 (1,23;2,90) |  | 49 (15.8) | 1,45 (0,87;2,42) |
| Yellow Fever vaccine |  |  |  |  |  |
| No | 29 (12.1) | 1 |  | 22 (9.2) | 1 |
| Yes | 128 (18.7) | 1,86 (1,19;2,93) |  | 98 (14.3) | 1,74 (1,00;3,01) |
| Hepatitis B vaccine |  |  |  |  |  |
| No | 35 (13.1) | 1 |  | 29 (10.9) | 1 |
| Yes | 107 (19.1) | 1,78 (1,16;2,75) |  | 80 (14.3) | 1,28 (0,76;2,17) |
| Nutrition |  |  |  |  |  |
| Breast feeding (length) |  |  |  |  |  |
| >= 4 months | 36 (18.9) | 1 |  | 18 (9.5) | 1 |
| < 4 months | 94 (16.6) | 1,00 (0,64;1,57) |  | 75 (13.3) | 1,14 (0,61;2,12) |
| Never | 80 (14.5) | 0,82 (0,52;1,29) |  | 8 (14.6) | 1,36 (0,73; 2,54) |
| BMI – (WHO) |  |  |  |  |  |
| No obesity | 177 (15.9) | 1 |  | 154(13.8) | 1 |
| Obesity | 33 (17.0) | 1,21 (0,78;1,87) |  | 20 (10.3) | 0,56 (0,32;0,96) |
| Physical activity level (High/Low) plus TV watching |  |  |  |  |  |
| High + ≤ 2 hours watching TV | 43 (14.7) | 1 |  | 37 (12.6) | 1 |
| High + > 2 hours watching TV | 36 (16.7) | 1,13 (0,68;1,89) |  | 28 (13.0) | 1,16 (0,63;2,13) |
| Low + ≤ 2 hours watching TV | 60 (17.0) | 1,20 (0,76;1,88) |  | 44 (12.5) | 1,03 (0,60;1,75) |
| Low + > 2 hours watching TV | 45 (14.2) | 0,97 (0,60;1,56) |  | 54 (17.0) | 1,59 (0,93;2,70) |
| Physical activity |  |  |  |  |  |
| High (at least 150 minutes per week) | 83 (15.9) | 1 |  | 67 (12.8) | 1 |
| Low (less than 150 minutes per week) | 111 (15.7) | 1,01 (0,73;1,40) |  | 100 (14.2) | 1,13 (0,77;1,66) |
| Time in front of TV |  |  |  |  |  |
| Up to 2 hours per day | 107 (16.2) | 1 |  | 81 (12.3) | 1 |
| More than 2 hours per day | 83 (15.2) | 0,92 (0,66;1,28) |  | 82 (15.0) | 1,38 (0,94;2,03) |
